# Supplementary material for: Human immunodeficiency virus 1 5′-leader mutations in plasma viruses before and after the development of reverse transcriptase inhibitor-resistance mutations
Source: J Gen Virol. 2023 Oct 6;104(10):001898. doi: 10.1099/jgv.0.001898 (PMC10721937; doi:10.1099/jgv.0.001898)
Supplement: Supplementary material 1 [file jgv-104-1898-s001.pdf]

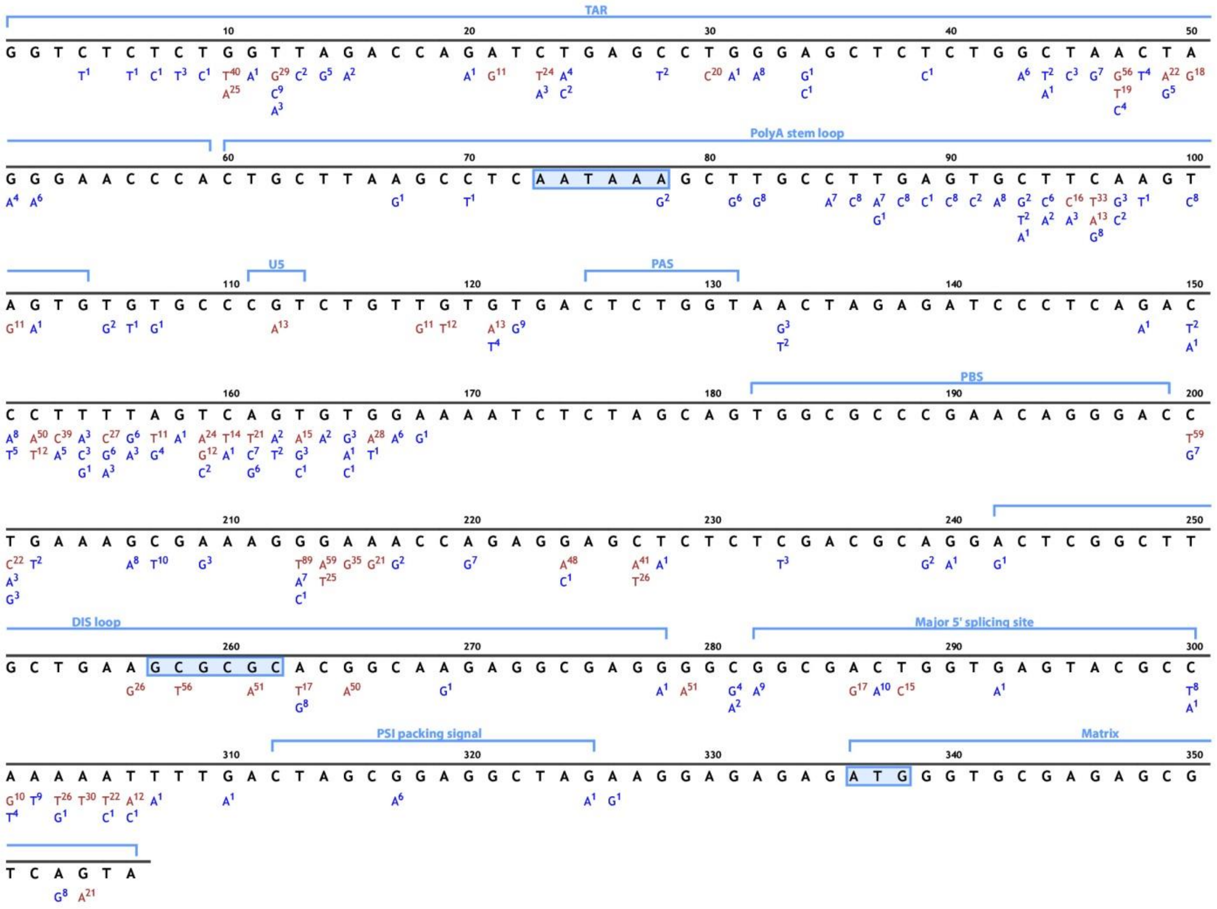

Fig. S1: HIV-1 5’-leader nucleotide differences from the HXB2 sequence observed in 1417 sequences in the Los Alamos National Laboratories HIV Sequence Database dataset. Superscripts indicate the percentage of times that a nucleotide was reported. Highlighted regions include (1) TAR, trans-activation response element; (2) poly(A), polyadenylation signal loop with a box surrounding the poly(A) motif; (3) U5; (4) PAS, primer activation signal; (5) PBS, primer binding site; (6) DIS, dimer initiation signal loop with a box surrounding the palindromic

DIS; (7) major 5’ splicing site; (8) psi packaging signal; (9) matrix protein with a box surrounding the start codon.

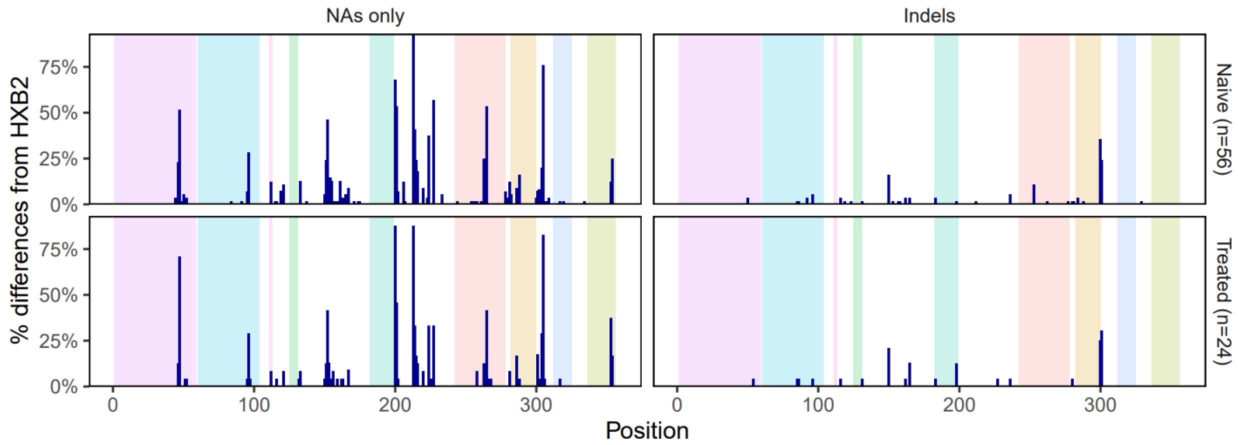

Fig. S2: Distribution of HIV-1 5’-leader nucleotide differences from HXB2 and indels in the 56 baseline sequences from ART-naïve individuals compared with the 24 baseline sequences from ART-experienced individuals.
